# Isotopic Nitrogen and Carbon Allocation Among Soybean Plant Parts Under Impact of *Bradyrhizobium japonicum* Strains

**DOI:** 10.3390/plants15121900

**Published:** 2026-06-18

**Authors:** Raminta Skipitytė, Rūta Barisevičiūtė, Yasha Jamil, Monika Toleikienė

**Affiliations:** 1 Institute of Agriculture, Lithuanian Research Centre for Agriculture and Forestry, Instituto al, 1, 58344 Akademija, Lithuania; yasha.jamil@lammc.lt (Y.J.); monika.toleikiene@lammc.lt (M.T.); 2 Center for Physical Sciences and Technology, Savanorių av. 231, 02300 Vilnius, Lithuania; ruta.bariseviciute@ftmc.lt

**Keywords:** Laulema, Merlin, seed inoculation, fertilization, cool climate, maturity group, organic farming

## Abstract

Understanding how plants regulate nitrogen (N) and carbon (C) allocation among their organs under adverse environmental and climatic conditions remains a significant challenge, despite its direct impact on the value of plant residues and agricultural products. Therefore, this study aimed to examine the dynamics of N and C through their stable isotope ratios in two soybean varieties of differing maturity groups (Merlin and Laulema) inoculated with various nitrogen-fixing *Bradyrhizobium japonicum* bacterial strains. The contents of N and C as well as their isotopic ratios in soybean plant parts were analyzed at full-flowering (R2) and full-maturity (R8) stages. The results demonstrated overall compatibility between soybean varieties and selected *B. japonicum* strains, resulting in up to 32 nodules per plant; however, significant variation in root nodule numbers was observed. From a physiological perspective, both the soybean variety and the strain of nitrogen-fixing bacteria significantly influenced nitrogen stable isotope ratios across different plant organs, including roots, shoots, stems, pods, and seeds, with similar trends in *δ*(^15^N) variation among plant parts observed in both varieties. In contrast, the main differences in carbon stable isotope composition were observed among varieties less affected by the amendment strategy. N content was higher in roots and shoots during flowering and declined by twofold in roots and fivefold in aboveground biomass at maturity, reflecting extensive nitrogen remobilization to support seed formation. From an agronomic perspective, the highest yields were achieved by the inoculated soybean Merlin, with more than 3 t ha^−1^. However, the positive effects of symbiosis can improve yields in less productive varieties like Laulema, making them comparable to those of more productive varieties. Soybean inoculation not only influenced the isotopic redistribution within the plant but also proved to be an effective practice for increasing seed N content, with strain AGF78 producing the highest number of nodules and a significantly high amount of nitrogen in seeds, followed by SEMIA5079, the least effective being RF10.

## 1. Introduction

Soybean (*Glycine max* (L.) Merr.) is primarily known as a nutritious food source for both humans and animals and is a crop with multiple purposes [1]. The Green Deal strategy encourages making protein crops more profitable and competitive in Europe [2], with soybean cultivation emerging as a promising option. While soybean production in cool regions is gaining attention, success depends on introducing adapted varieties with improved yield performance and management practices tailored to lower temperatures and long-day conditions [3,4,5,6].

The adoption of legume-based cropping systems and the development of biofertilizers that exploit biological nitrogen fixation (BNF) are essential strategies for sustainable agriculture, as they enhance soil health and productivity by increasing N and other nutrient availability, disrupting pest cycles, stimulating soil microbial activity [7,8], and mitigating the adverse effects of intensive fertilizer use [8,9]. Various strategies have been employed to enhance biological nitrogen fixation [10,11,12,13]. A study by Skipitytė, Barisevičiūtė and Toleikienė [6] demonstrated that inoculation is an effective practice for improving productivity parameters in cool climates. Such symbiotic relationships are particularly important in soils that naturally lack nitrogen-fixing bacteria.

To maximize the N-fixing potential of soybeans, it is essential to select *Bradyrhizobium japonicum* strains that are well adapted to cool climates and soil conditions—particularly in regions like Lithuania, where native populations of *B. japonicum* are absent [6,14,15]. This symbiotic relationship can be sufficient and reduce dependence on artificial fertilizers and offer environmental and economic benefits [3,16]. Bacteria reside within the root nodules and convert atmospheric N_2_ into ammonium (NH_4_^+^), making it available for plant uptake [17]. However, symbiotic bacteria can alter internal nutrient flows and, consequently, isotopic fractionation within the plant. The degree of this fractionation depends on the specific bacterial strain and the host plant species [18,19]. In turn, nitrogen isotope composition can serve as an indicator of nitrogen cycling processes within plants, as nitrogen isotope discrimination—reflecting a preference for lighter isotopes during biological reactions—is influenced by shifts in nitrogen supply or demand. Nitrogen isotope discrimination *Δ*(^15^N) relative to the source can be calculated when the substrate and the product isotopic values are known [20,21].

To grow efficiently and withstand stress, plants must use their available resources strategically. Among these resources, N and C are especially important as they form the foundation of plant metabolism. Through photosynthesis, plants take in carbon dioxide (CO_2_) and convert it into non-structural carbohydrates (NSCs), which serve as vital energy sources and essential building blocks for processes such as growth, defense, and interactions with other organisms [22]. At the same time, N acquired from the soil or through symbiotic fixation supports the synthesis of amino acids, proteins, and other key compounds that enable these metabolic activities. Together, the dynamics of these essential elements shape a plant’s ability to develop, adapt, and thrive [23].

Plants distribute C in ways that enhance growth, reproduction, and competitive success within plant communities. Allocation patterns vary among environments and reflect different life forms, resulting in distinct C allocation strategies [24]. The balance between C supply and N demand can affect nodulation, N fixation rates, biomass production, and yield. High soil N availability generally reduces C allocation to nodules and suppresses BNF, whereas N-limited conditions promote greater C investment in nodules and increased symbiotic activity [25]. Environmental factors such as temperature, light, drought, and nutrient availability further modify allocation patterns and symbiotic efficiency [26,27,28].

Stable isotope ratios are primarily used as indicators of source materials and can also reflect the reactions in which they participate. Although in many agronomic studies they are applied as indicators of nitrogen derived from the atmosphere, their role in elemental allocation can be more complex and may depend on various environmental and physiological factors [29,30]. Different varieties exhibit genotypic variation in allocation strategies, suggesting opportunities for breeding varieties with improved N-use efficiency and enhanced seed protein yield; therefore, efficient coordination between C supply and N acquisition is critical for maximizing soybean productivity. Of the two varieties, Merlin is more productive than Laulema, though it produces smaller seeds. Therefore, the application of appropriate management practices is expected to yield more promising results for the less productive varieties [6]. While genotypic variation is well documented, how these differences manifest in cool environments beyond the species’ typical northern distribution boundary remains poorly understood.

Organic farming of soybeans can benefit soil health by improving many properties, including soil organic matter, available N, soil microbial diversity, and soil C and N transformation rates. Furthermore, organic systems can be as effective as conventional treatments, even under continental semi-arid conditions [31]. The research hypothesis was that the interaction between soybean plants and specific *B. japonicum* strains may change the accumulation of C and N and their isotopic ratios, as well as the redistribution of these elements within the plant parts. Therefore, this study aimed to evaluate the effect of inoculation on the development of two soybean varieties that are promising for organic cultivation, Merlin and Laulema. To achieve this aim, we examined the distribution of nitrogen and carbon isotopic ratios, along with their elemental contents—focusing primarily on nitrogen—across different plant developmental stages. These patterns were evaluated across various nitrogen-fixing strains and compared against both non-inoculated and fertilized controls.

## 2. Results

### 2.1. Effects of Treatment on Nitrogen Stable Isotope Ratio and N Content in Different Plant Parts at Flowering and Full-Maturity Stages

The *B. japonicum* strain and fertilization significantly influenced isotopic and elemental distribution in the plant organs of Laulema and Merlin at the full-flowering stage (R2). Nodules formed in various numbers in inoculated plants exceptionally, demonstrating in general the compatibility between the inoculants and the soybean varieties. However, nodule number varied among strains, resulting in up to 32 nodules per plant; the highest count was observed in Merlin inoculated with strain AGF78 and the lowest in Laulema inoculated with RF10. This trend in nodule abundance was consistent across both varieties, with AGF78 being the most effective, followed by SEMIA5079 and RF10. Despite these differences in nodule number, nodule dry weight did not differ significantly among treatments (Table 1).

The analysis revealed statistically significant differences in *δ*(^15^N) values within the shoots at the flowering stage, whereas root *δ*(^15^N) values remained unaffected by the treatments. In shoots, inoculated treatments generally exhibited lower nitrogen isotopic ratios, except for inoculant RF10, which showed even higher nitrogen stable isotope ratio values than the control during flowering, and this trend was observed in both varieties. Across all treatments, shoots consistently displayed higher *δ*(^15^N) values than roots; however, this root-to-shoot discrimination against the lighter isotope was less pronounced in inoculated plants compared to the control group.

**Figure 1:**
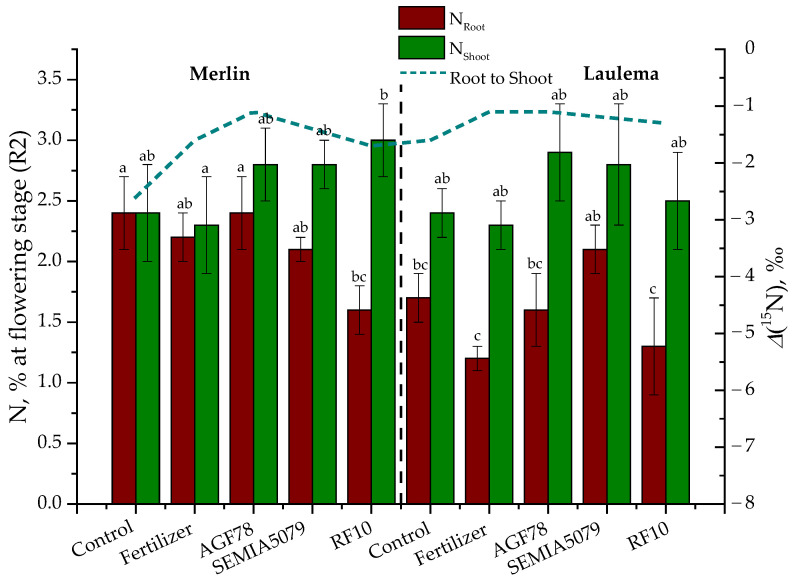
Nitrogen content in roots and shoots at flowering stage (R2) and isotopic root-to-shoot discrimination. Means followed by the same letters in roots or shoots do not differ significantly from one another (one-way ANOVA, *p* ≤ 0.05).

Shoot N content did not differ significantly between the two soybean varieties across the treatments. The only exception was Merlin plants treated with RF10, which showed the highest shoot N content. Roots of Laulema contained a significantly lower N content than those of Merlin, resulting in a more distinct N content between shoots and roots in Laulema (Figure 1).

**Figure 2:**
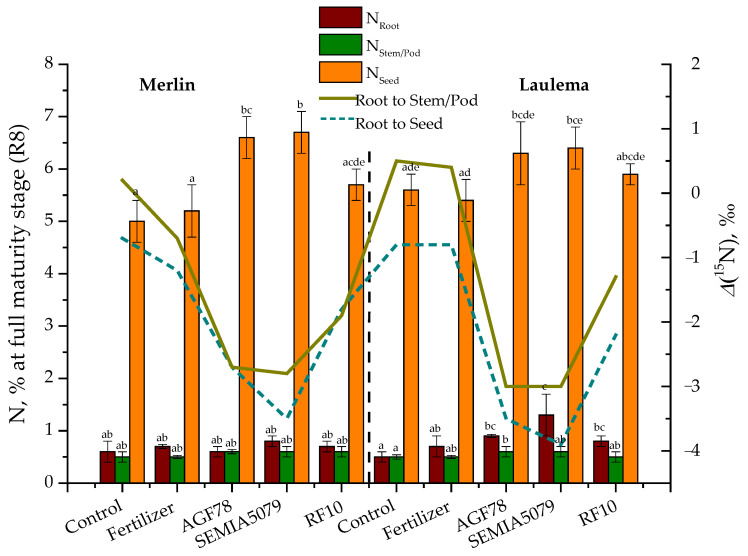
Nitrogen content in roots, stems and pods, and seeds at full-maturity stage and isotopic root-to-stem/pod and stem/pod-to-seed discrimination. Means followed by the same letters in roots, stems/pods, and seeds do not differ significantly from one another (one-way ANOVA, *p* ≤ 0.05).

At full maturity, the pattern of ^15^N/^14^N ratios differed markedly (Table 2). In roots, nitrogen stable isotope ratios were higher in inoculated plants compared with the control and fertilized treatments, with the strongest effects observed for strains AGF78 and SEMIA5079. In contrast, stems and pods showed significantly lower nitrogen isotope ratios under inoculated treatments, indicating either a shift in nitrogen source or changes in isotopic discrimination. A similar trend was observed in seeds, where the isotopic difference between control and inoculated treatments was even more pronounced. At full maturity, both the direction and magnitude of isotopic discrimination between roots and stems/pods, as well as between roots and seeds, were different; the range of the magnitude changed from *Δ*(^15^N) values of −1.5‰ in Merlin and 0.5‰ in Laulema at flowering to 3.0‰ and 3.5‰ at maturity, respectively. The discrimination was more pronounced in the inoculated treatments, particularly SEMIA5079 and AGF78, compared with the control and fertilized treatments. This consistent trend across both varieties underscores the impact of inoculation on isotopic allocation, even when the nitrogen content itself did not show significant differences (Figure 2). 

#### 2.1.1. Effects of Variety and Amendment on Nitrogen Isotopic Signatures at Flowering and Full-Maturity Stages

In this section, the effects of amendment (*B. japonicum* strain or organic fertilizer), variety (Merlin and Laulema), and their interaction on isotopic signatures at the flowering and full-maturity stages were evaluated. In roots, no statistically significant differences were observed in nitrogen stable isotope ratios; however, a slight trend toward higher values was noted in treatments involving strain RF10, a characteristic shared by both varieties.

Conversely, both the amendment type and variety significantly impacted shoot nitrogen stable isotope values. A two-way ANOVA revealed a significant effect of amendment on *δ*(^15^N) values (F_4,36_ = 6.97, *p* < 0.001) and a significant effect of variety (F_1,36_ = 6.48, *p* = 0.015). The interaction between *B. japonicum* strain and variety was not significant (F_4,36_ = 1.02, *p* = 0.41). Therefore, amendment with *B. japonicum* strains explained a larger proportion of the variance in *δ*(^15^N) than variety, with no observed interactive effects. In Merlin, the control exhibited higher nitrogen isotopic ratios compared to the other treatments, except for strain RF10. This tendency was also present in Laulema, though to a lesser extent, highlighting physiological and developmental differences between the varieties.

At full maturity, the isotopic differences between treatments were even more pronounced. Roots exhibited opposite isotopic trends relative to the stems, pods, and seeds. In roots, a significant effect of *B. japonicum* strain on *δ*(^15^N) values (F_4,30_ = 12.64, *p* < 0.001) was found. In contrast, neither variety (F_1,30_ = 2.02, *p* = 0.166) nor the amendment with the *B. japonicum* strain and variety interaction (F_4,30_ = 1.45, *p* = 0.242) had a significant effect on root *δ*(^15^N) values.

As with the roots, a highly significant effect of treatment was observed on *δ*(^15^N) values in soybean stems and pods (F_4,30_ = 27.33, *p* < 0.001). However, neither variety (F_1,30_ = 0.02, *p* = 0.901) nor the interaction between the amendment and variety (F_4,30_ = 1.98, *p* = 0.123) had a significant effect.

In contrast, significant effects of *B. japonicum* strain (F_4,30_ = 58.37, *p* < 0.001) and variety (F_1,30_ = 42.65, *p* < 0.001) on *δ*(^15^N) values were found in soybean seeds. In addition, a significant interaction between *B. japonicum* strain and variety was observed (F_4,30_ = 2.90, *p* = 0.039), indicating that the *δ*(^15^N) response to the amendment differed between soybean varieties. These results suggest strong amendment- and variety-specific differences in N acquisition and allocation to seeds, particularly when compared with the patterns observed in roots, stems, and pods.

In summary, while seed isotopic signatures were influenced by the strain, variety, and their interaction, the isotopic profiles of roots, stems, and pods were solely affected by the amendment, highlighting organ-specific nitrogen isotope responses.

#### 2.1.2. Accumulated Nitrogen and Effects of Variety and Amendment on N Content

The accumulation of N and C across different developmental stages and soybean plant organs served as a vital indicator of nitrogen fixation efficiency, nutrient transport, and carbon incorporation (Table 3 and Table 4).

**Figure 3:**
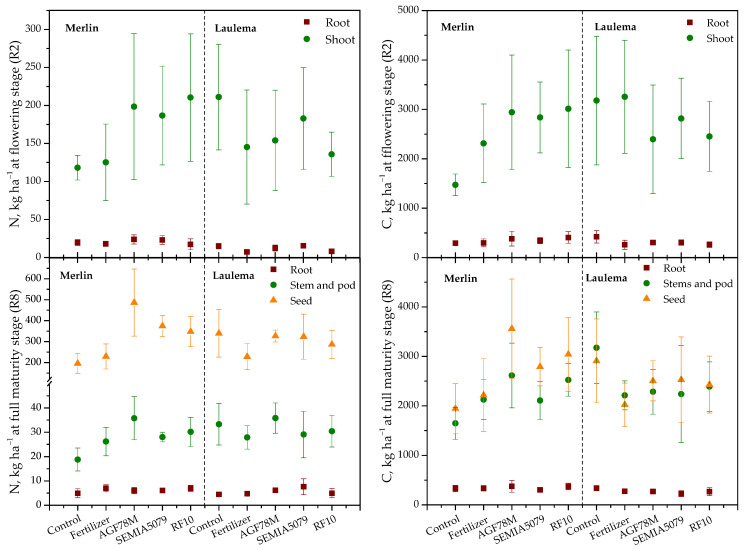
Nitrogen and carbon content in roots, shoots, stems and pods, and seeds at flowering and full-maturity stages.

The greatest variation in accumulated nitrogen was observed in the roots at the flowering stage, with the highest values recorded in plants treated with SEMIA5079 (23.1 ± 5.80 kg dry weight (DW) N ha^−1^ and 15.3 ± 1.66 kg DW N ha^−1^ in Merlin and Laulema, respectively). Roots of fertilized plants and those treated with strain RF10 accumulated the lowest amounts of nitrogen in both soybean varieties. However, none of the factors significantly affected total nitrogen in the shoots or carbon accumulation per hectare in the roots, stems, or shoots. Nonetheless, the mean values suggested a positive influence of inoculation with *B. japonicum*, with notable differences among strains (Table 3, Figure 3).

At full maturity, root nitrogen values were generally uniform, with differences among treatments primarily evident in seeds. Seed nitrogen content was significantly higher in Merlin when inoculated with strain AGF78. In Laulema, fertilization generally had a negative effect on total seed nitrogen accumulation, while inoculation did not significantly increase nitrogen content, although there was a slight upward trend. In contrast, carbon data for Laulema suggest an opposite response to inoculation; however, these differences were not statistically significant due to high variability (Table 4, Figure 3).

A two-way ANOVA revealed significant effects of *B. japonicum* strain (F_4,25_ = 9.13, *p* < 0.001) and variety (F_1,25_ = 54.58, *p* < 0.001) on N content in soybean roots at flowering stage. In addition, a significant interaction was observed between *B. japonicum* strain and variety (F_4,25_ = 4.71, *p* = 0.006), indicating variety-specific responses to the amendment.

A significant effect of *B. japonicum* strain on N concentration in aboveground soybean biomass was observed at the flowering stage (F_4,36_ = 4.53, *p* = 0.005). In contrast, neither variety (F_1,36_ = 0.92, *p* = 0.345) nor the interaction between *B. japonicum* strain and variety (F_4,36_ = 1.45, *p* = 0.237) had a significant effect. These results indicate that the *B. japonicum* strain had a stronger impact on N concentration in aboveground biomass than varietal differences.

Significant effects of *B. japonicum* strain (F_4,29_ = 8.15, *p* < 0.001) and variety (F_1,29_ = 12.28, *p* = 0.002) on N concentration in soybean roots at harvest were revealed. In addition, a significant *B. japonicum* strain and variety interaction was observed (F_4,29_ = 5.24, *p* = 0.003), indicating that N concentration in roots responded differently to inoculation treatments depending on the soybean variety. Meanwhile, a two-way ANOVA showed a significant effect of *B. japonicum* strain on N content in soybean stems and pods at harvest (F_4,30_ = 5.18, *p* = 0.003). In contrast, neither variety (F_1,30_ = 1.81, *p* = 0.189) nor the interaction of amendment and variety (F_4,30_ = 0.67, *p* = 0.619) had a significant effect.

The most pronounced differences between amendments were observed in the seeds (F_4,30_ = 18.40, *p* < 0.001). In contrast, neither variety (F_1,30_ = 0.41, *p* = 0.527) nor the amendment and variety interaction (F_4,30_ = 1.86, *p* = 0.144) had a significant effect. Across plant organs at harvest, the *B. japonicum* strain consistently influenced N concentration, whereas varietal effects were organ-specific and most evident in the roots. Furthermore, interaction effects were primarily significant for root N concentration.

### 2.2. Variation in Carbon Among Soybean Varieties

The carbon stable isotope ratios, carbon content and carbon-to-nitrogen ratio (C/N) in different plant parts of the soybean varieties Laulema and Merlin at the flowering and full-maturity stages are provided in Table 5 and Table 6.

Carbon dynamics revealed distinct patterns across maturity stages and plant organs, reflecting shifts in allocation and nutrient coupling during plant growth. At the flowering stage, carbon isotopic composition was largely uniform between below- and aboveground tissues, suggesting minimal isotopic fractionation during carbon assimilation and translocation. During this phase, differences among amendments and varieties were negligible—though *δ*^13^C values in Merlin shoots were slightly higher—indicating that early carbon partitioning was primarily governed by plant developmental status rather than management practices.

As plants progressed to full maturity, clearer contrasts emerged among organs and varieties. While carbon concentrations in roots, stems, and pods remained comparable to those observed at flowering, their C/N ratios increased markedly, pointing to increased nitrogen depletion associated with nitrogen remobilization to seed.

Meanwhile seeds were characterized by high carbon concentrations and consistently low C/N ratios, confirming their role as the primary sink for both carbon and nitrogen during the reproductive phase. Additionally, seeds were isotopically more depleted in ^13^C compared with vegetative organs, suggesting preferential allocation of newly assimilated or metabolically fractionated carbon during seed filling.

Although neither inoculation nor fertilizer substantially altered total carbon accumulation, both influenced carbon–nitrogen relationships within plant tissues. Inoculation with *B. japonicum* strains resulted in lower C/N ratios in the roots and stems compared to the control and fertilized treatments, suggesting more balanced coordination between carbon retention and nitrogen availability. Overall, carbon allocation patterns were strongly organ-specific and developmentally regulated, with microbial inoculation primarily affecting nutrient balance rather than total carbon storage.

Regarding the effects of variety and amendment and their interaction on carbon measurements, a significant effect of variety on *δ*(^13^C) values was evident at both development stages and across all the investigated plant parts. However, it was less pronounced in the roots at the flowering stage but highly significant in the seeds (F_1,28_ = 75.21, *p* < 0.001). In the seeds, *δ*(^13^C) values were influenced by both variety and the interaction between variety and amendment (F_4,28_ = 7.22, *p* < 0.001), indicating that the carbon isotopic response to the treatments differed between the two soybean varieties. In contrast, the main effect of the amendment was not significant (F_4,28_ = 1.40, *p* = 0.261).

### 2.3. Productivity Parameters of Inoculated and Non-Inoculated Soybeans

Agronomic parameters for the inoculated and non-inoculated soybeans are presented in Table 7. The highest seed yields were obtained from Merlin, reaching an average of 315 g m^−2^ or 3150 kg ha^−1^. However, when inoculated, Laulema achieved yields comparable to those of Merlin, particularly when treated with strain SEMIA5079. The primary differences in 1000-seed weight were observed between the varieties. In general, Merlin produces smaller seeds than Laulema; however, inoculation tended to increase seed size in both. The most pronounced improvements were observed in seed protein content, where statistically significant increases were detected in inoculated plants, particularly those treated with strains SEMIA5079 and AGF78, compared to the control and fertilized treatments.

## 3. Discussion

The isotopic variation in plant tissues indicates sources of nitrogen in the plant’s growing environment. Legumes that obtain most of their nitrogen from atmospheric N_2_ tend to have relatively low *δ*(^15^N) values around 0‰, similar to the source, whereas plants fertilized with organic fertilizers generally have higher and more variable isotopic signatures. In some cases, like seabird guano, which is a primary sources of nitrogen for plants, *δ*(^15^N) values in plant tissues may reach 20‰ or even higher [32]. Typically, animal- and plant-based organic fertilizers can be separated as they have distinct isotopic values [33]. Unfortunately, the *δ*(^15^N) values of mineral fertilizers are close to those of atmospheric nitrogen, and these nitrogen sources cannot be separated by the isotopic natural abundance technique [34]. Isotopic differences between different varieties of the same plant species can also arise from genetic, physiological, and ecological differences that affect how plants acquire and process elements such as carbon and nitrogen. Different genetic backgrounds can influence photosynthetic efficiency, enzyme activity, root architecture, and nutrient uptake strategies and lead to variety-specific isotope fractionation, even under identical conditions [35]. External factors like agricultural practices (e.g., intercropping) may cause a significant redistribution of elements such as carbon and nitrogen in plants [36]. In our study, the overall isotopic range of measured *δ*(^15^N) values in plant tissues was more than 5‰ (from −0.3 to 5.7‰), whereas *δ*(^13^C) exhibited a range of about 3‰ (from −28.1 to −24.7). The nitrogen stable isotope ratios within treatments in the same plant parts were in the range of ~1–2‰. Stable isotope ratios varied in relatively small ranges, but keeping in mind the natural abundance stable isotope ratios used in this study, the effects of inoculation were significant and sufficient to distinguish between treatments.

Stable isotope ratios in the plant samples were measured at flowering and full-maturity stages. The flowering stage represents a critical period during which root function and symbiotic activity play a central role in determining subsequent plant growth and yield potential. During the flowering stage (R1–R2), the soybean root system is well developed and physiologically active, supporting both vegetative growth and the onset of reproductive development [37]. At this stage, root nodulation is abundant, and nodules are typically pink or reddish in color, indicating active biological nitrogen fixation. Nitrogen fixation rates are generally at or near their maximum during flowering, supplying a substantial proportion of the plant’s nitrogen demand [38]. Thus, variation in nitrogen isotopic ratios can be used to trace nitrogen sources and sinks within the plant and provide insights into how plants distribute the elements to meet the needs of various organs. In our study, the most effective nodulation was observed in the cases of strains AGF78 and SEMIA5079 (Table 1). Despite the fact that the nodule weight did not differ significantly, the nitrogen stable isotope ratio tended to be lower in cases with more nodules. Efficient nodulation is crucial for ensuring N_2_ fixation, highlighting that optimized inoculation practices may offer a sustainable strategy to meet soybean’s N demands during the growing season when weather conditions challenge growth dynamics. During the 2024 growing season, the mean air temperature was higher than the long-term average, providing favorable conditions for soybean growth; additionally, precipitation was adequate during the period of rapid plant development.

At the flowering stage, nitrogen stable isotope ratios and nitrogen content changed in response to the application of *B. japonicum* strains and showed the redistribution of nitrogen within the plants’ parts at different development stages. Across plant parts, shoots consistently showed higher *δ*(^15^N) values than roots, suggesting that N allocated to aboveground tissues was enriched in ^15^N relative to the roots, reflecting either fractionation during root-to-shoot N translocation or changes in sourcing. Meanwhile, the nitrogen content was 1.9% on average in roots, and it was 2.6% on average in shoots. However, strain RF10 had rather a negative effect on root nitrogen content. This may be explained by the lower compatibility between the strain and the host plant, resulting in reduced nitrogen uptake and consequently decreased nitrogen accumulation in plant tissues. Notably, shoot nitrogen content did not increase significantly. Among the three selected *B. japonicum* strains, SEMIA5079 is a widely used and well-studied soybean inoculant strain, particularly in South American countries like Brazil [39]. It is known for its high efficiency in establishing symbiosis with soybean roots and its strong capacity for biological nitrogen fixation (BNF) and promotion of better yields. Meanwhile, there is considerably less information on the other strains like AGF78 [35,40] and RF10, also named Rhizofix in the other studies [41,42], and their impact on nitrogen dynamics in plants growing in cool climates.

Therefore, at full maturity, the pattern of nitrogen dynamics differed markedly from that observed at flowering. The root N content in most treatments was below 1%, about half of the values recorded at the flowering stage (~2%). A significantly higher nitrogen content was observed in inoculated cases, but only for Laulema. The N content in stems and pods was also low, in most cases around 0.5%, which is about five times lower than in aboveground biomass at the flowering stage. N content was highest in seeds and tended to be higher in inoculated treatments. In roots, nitrogen stable isotope ratios were higher in inoculated treatments than in the control, with the greatest increase occurring in plants inoculated with strains AGF78 and SEMIA5079. Roots at maturity showed higher *δ*(^15^N) values than at the flowering stage, showing the ongoing dynamics of nitrogen circulation in the plant. In contrast, stems and pods of inoculated plants showed significantly lower nitrogen isotope ratios (by approximately 0.6 to 1.5‰) compared with the control, indicating a greater contribution of nitrogen derived from biological nitrogen fixation (BNF); however, accurate quantification of these proportions using the ^15^N natural abundance method remains challenging and was not performed in this study [43]. A similar trend was observed in seeds, where differences in nitrogen isotope values between control and inoculated plants were even more pronounced. Notably, inoculated soybeans (especially those treated with AGF78 and SEMIA5079) accumulated substantially more nitrogen in their seeds. The large differences in *δ*(^15^N) values in these plants reflect not only enhanced BNF but also a higher overall amount of nitrogen accumulated in seeds and, in the case of Laulema, in the roots.

Seeds are highly demanding because they act as the primary metabolic sink, requiring intense accumulation of carbohydrates for energy and amino acids for storage to ensure successful seedling and survival. Therefore, seeds accumulated the majority of plant N (5–6.7%), with inoculation (AGF78 and SEMIA5079) increasing seed N content. Stems and pods consistently had the lowest *δ*(^15^N) and N content, acting primarily as transport tissues. Both seeds and aboveground biomass in inoculated treatments had lower nitrogen stable isotope ratios compared with roots, showing the nitrogen source with the isotopic signal expected for BNF. Some studies argue that biological nitrogen fixation is insufficient under certain conditions and that additional fertilization is needed [44]. However, organic granulated cattle manure fertilizer had a surprisingly lower impact on seed N content for both varieties (Table 2, Figure 2), indicating that rhizobial inoculation more effectively enhanced N allocation to reproductive tissues, particularly at late maturity.

While nitrogen isotopic ratios in plant tissues are mainly influenced by the relative contributions of soil nitrogen and biological nitrogen fixation and can be further modulated by fractionation during uptake and assimilation, the carbon stable isotope ratio derived from atmospheric CO_2_ is primarily regulated by water-use efficiency (WUE)—a key factor determining plant productivity under limited water supply, defined as the ratio of carbon assimilation to water transpiration at the leaf level. In agronomic terms it is the ratio between dry matter (harvest) produced and water used (applied), and this process is more pronounced in arid areas with limited water supply [45]. However, improving WUE could reduce adverse effects of drought, which is important in changing environments and improves productivity. Therefore, it can be important to know the physiological differences between varieties related to WUE. Carbon measurement results suggest that varietal differences influenced carbon isotope discrimination, while nitrogen management practices had little effect on *δ*(^13^C) at both flowering and maturity stages, except in the case of seeds, where a strong varietal effect on seed *δ*(^13^C) indicates differences in carbon assimilation or water-use efficiency between soybean varieties, while the significant interaction suggests that management practices modulated these differences in a variety-dependent manner.

Relative humidity and water availability regulate the opening of leaf stomata, thereby controlling CO_2_ uptake and transpiration and ultimately influencing the carbon isotopic composition of plant tissues. During periods of rapid growth, increased CO_2_ demand can alter the ratio of intercellular to atmospheric CO_2_, respiratory CO_2_ fluxes, and the reduction in ^13^C fractionation during photosynthesis, leading to variation in the plant’s carbon isotopic signature. Merlin matures later, and its prolonged development period likely accounts for the observed lower ^13^C/^12^C ratio in its tissues during the flowering stage compared to Laulema. During maturity, Laulema maintained higher *δ*(^13^C) values in its tissues. Given the expectation that plants that accumulated more carbon would exhibit higher *δ*(^13^C) values (reflecting lower ^13^C fractionation during photosynthesis), the observed patterns were surprising. In Merlin, both control and fertilized plants accumulated lower amounts of carbon, and their tissues were expected to be most depleted in ^13^C. However, this was not the case. In fact, Merlin soybeans—which accumulated more carbon (reaching 30–45% in seeds)—showed a tendency toward lower ^13^C/^12^C ratios (Figure 3), indicating stronger isotopic fractionation during photosynthesis.

Meanwhile, seeds were characterized by high carbon concentrations and consistently low C/N ratios, demonstrating their role as the dominant sink for both carbon and nitrogen during the reproductive phase. In addition, seeds were isotopically more depleted in ^13^C compared with vegetative organs, indicating preferential allocation of newly assimilated or metabolically fractionated carbon during seed filling. Overall, carbon allocation patterns were strongly organ-specific and developmentally regulated, with microbial inoculation primarily affecting nutrient balance rather than total carbon storage. Laulema plants in the control treatment accumulated the greatest amount of carbon (Figure 3); however, they did not exhibit higher *δ*(^13^C) values (except for roots). Despite their presumably higher photosynthetic rates, their *δ*(^13^C) values were similar to those of inoculated plants, which accumulated 15–16% less carbon in seeds (Figure 3). These results suggest that carbon accumulation does not always directly predict *δ*(^13^C) values, highlighting complex interactions between carbon assimilation, stomatal behavior, and isotopic fractionation under different treatments.

Many studies focus on soybean productivity and yield [3,15,46]. Agronomical parameters are important when choosing growing strategies. The soybean varieties Laulema and Merlin differ mainly in maturity, productivity, and response to inoculation under cool European conditions. Recent studies [3,6] showed that Laulema, an earlier-maturing variety, can reach seed yields of more than 2100 kg ha^−1^ under effective treatments. In contrast, Merlin, a slightly later-maturing variety, can achieve up to ~3000 kg ha^−1^, consistently outperforming Laulema. Moreover, Merlin tends to have a greater biomass and grain weight, while both varieties show improved nitrogen fixation when inoculated, although the magnitude of response is variety-dependent. Therefore, our study demonstrates the potential of soybean cultivation in cool climates of northern regions and confirms that inoculating soybean in new soils lacking effective Bradyrhizobium strains with commercial products is essential for ensuring nitrogen fixation through BNF rather than relying on N fertilizers.

Future studies should focus on investigating the translocation of N from the vegetative biomass to the harvestable grain as well as on the quantification of litterfall losses throughout the cropping season. These studies would further advance the quantification of BNF-N contributions to cropland N balances and account for the timing of additional losses due to residue mineralization [47].

## 4. Materials and Methods

### 4.1. Study Site and Experimental Design

The experiment was conducted in organically managed (no artificial fertilizers or pesticides) fields at the Lithuanian Research Centre for Agriculture and Forestry in Akademija (55.400516° N, 23.865672° E) in 2024. The field trial was established using a randomized complete block design with four replicates to improve statistical robustness and reduce the influence of field heterogeneity. Individual plots measured 1.5 m in width and 5.0 m in length, covering an area of 7.5 m^2^. The experimental field was characterized by a loamy Endocalcaric Epigleyic Cambisol (Drainic, Loamic; CM-can.glp dr.lo). The topsoil layer (0–25 cm) exhibited a pH of 7.5 and contained 0.077 g kg^−1^ of available P_2_O_5_, 0.138 g kg^−1^ of potassium, and 2.4% humus.

Two selected varieties of soybean—Laulema (very early-maturity group (0000)) and Merlin (early-maturity group (000)) were tested for their performance in the field along with control and granulated chicken manure fertilizer treatments whose use is allowed in organic fields. Soybeans were sown on 15 May. Five plants were collected from each plot at the flowering stage in July, and the crop was harvested from an area of 50 × 50 cm (0.25 m^2^) per plot in September, depending on the variety: 9 September for Laulema and 24 September for Merlin. A Kubota seeding machine (Kubota GmbH, Rodgau, Germany) was used.

Agronomic parameters were assessed using samples collected from four 0.25 m^2^ area subplots for each treatment. Five plants were selected from each plot, and their measurements were averaged. For stable isotope and elemental content analyses, five plants per sample were pooled and homogenized prior to analysis. To assess nitrogen and carbon accumulation, mean values per plant were calculated from measured nitrogen and carbon percentages, assuming a plant density of 800,000 plants per hectare.

The soybean seeds were inoculated with products (applied and air-dried) containing nitrogen-fixing bacteria (1 × 10^9^ colony-forming units) prior to sowing. For the control, one treatment was not inoculated and received no fertilization and another was not inoculated but fertilized with organic manure (granulated manure) at a rate of 45 kg ha^−1^ N. The experimental design is explained in the following table (Table 8).

### 4.2. Weather Conditions

**Figure 4:**
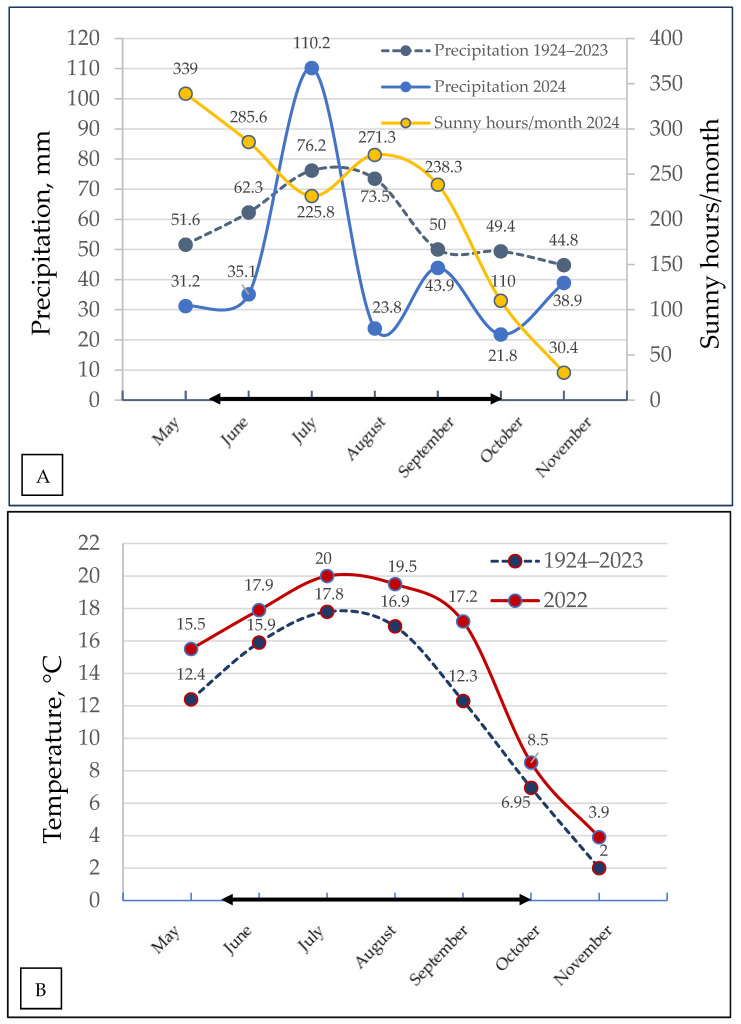
Weather conditions ((**A**) precipitation, and sunny hours per month, (**B**) temperature) in the study site in 2024 compared with a long time average (1924–2023) (temperature and precipitation). The sowing and harvesting periods are marked by the arrows.

Lithuania has a temperate climate characterized by warm summers, cold winters, and moderate precipitation throughout the year. During the 2024 growing season, the mean air temperature was 2.4 °C above the long-term average; however, it was still lower than the overall optimum for soybeans. Meanwhile, total precipitation was 63.3 mm lower than the multi-year average and not evenly distributed during the growing season; thus, the crop vegetation period in 2024 was warmer and drier than usual (Figure 4).

Soybeans grow best in warm, moderately humid climates; therefore, the dry summer created unfavorable conditions for plant growth. Therefore, Lithuania’s temperate maritime–continental climate, with moderately warm summers, cool nights, and variable rainfall, creates both opportunities and challenges for crops like soybean that require sufficient heat and moisture during the growing season.

### 4.3. Stable Isotope Analysis

Plant samples consisting of five plants collected from each of the four replicates were dried and homogenized prior to analysis. Stable isotope as well as percentage of C and N measurements were performed using the FlashEA 1112 elemental analyzer (Thermo Fisher Scientific (Delft, The Netherlands)) and the Thermo Fisher Scientific Finnigan Delta Advantage isotope ratio mass spectrometer (Thermo Fisher Scientific Finnigan Delta Advantage (Bremen, Germany)) system. Stable isotope ratio measurements were expressed relative to a standard: atmospheric air N_2_ and V-PDB for nitrogen and carbon, respectively, and expressed in delta (δ) notation, reported in parts per thousand (‰):

*δ*X = (R_sample_/R_standard_ − 1) × 10^3^

where R = ^15^N/^14^N or ^13^C/^12^C in the sample and in the standard.

During the analysis, samples were interspersed with several replicates of laboratory reference materials, calibrated against international reference materials, including IAEA-N-1, IAEA-600, provided by the International Atomic Energy Agency (IAEA), and USGS24, provided by the National Institute of Standards and Technology (NIST). The long-term standard deviation was <0.2‰; however, a subset of the soybean samples (~10%) was measured in duplicate to assess sample homogeneity, and the resulting values were pooled.

### 4.4. Statistical Analysis

The data were tested for normality and presented as means ± SDs. A one-way ANOVA was used to test the effect of treatment; meanwhile, a two-way ANOVA was performed to analyze how the data were affected by: amendment—different treatments (e.g., fertilization, inoculation with different commercially available *Bradyrhizobium japonicum* strains, and control); variety—soybean varieties (Merlin and Laulema); and amendment and variety interaction—whether varieties responded differently to amendments (see Table 8). Statistical analyses were performed using R Commander (version 4.3.3). Differences were regarded as statistically significant when *p* ≤ 0.05.

## 5. Conclusions

Inoculation with *Bradyrhizobium japonicum* strains resulted in effective nodulation and influenced different nitrogen distribution in different plant parts, particularly at maturity. The type of amendment and the strain of *B. japonicum* explained a larger proportion of the variance in *δ*(^15^N) than the soybean variety itself. In contrast, variety accounted for a larger proportion of the variance in *δ*(^13^C), which remained largely unaffected by inoculation or fertilizer, reflecting inherent physiological and development differences between the varieties. Therefore, the analysis of natural abundance stable isotope ratios proved to be a robust and informative approach for understanding the internal allocation of nitrogen and carbon in soybean plants.

At maturity, strong amendment- and variety-specific differences were observed in nitrogen acquisition and allocation to seeds, whereas minimal effects were noted regarding C and N content in the roots, stems and pods. Roots, stems, and pods exhibited elevated C/N ratios at maturity, highlighting nitrogen depletion associated with senescence and translocation to reproductive organs, while carbon was primarily retained in the structural biomass.

Inoculation consistently influenced nitrogen concentration across all plant organs at the full-maturity stage. The most pronounced differences between the amendments were observed in soybean seeds, where the N content increased by about 12–13% in Laulema and 24–25% in Merlin, with strains AGF78 and SEMIA5079 being the most effective. Consequently, inoculation enhanced the yields of the less productive variety, Laulema, making them comparable to those of the more productive Merlin.

These findings suggest that strategic inoculation can bridge the yield gap between varieties, facilitating the expansion of organic soybean cultivation in cool–temperate regions where nitrogen availability often limits productivity.

The superior performance of strains AGF78 and SEMIA5079 suggests that they are well adapted to the climatic conditions of Northern Europe, making them the cornerstone of agroecological practices, providing a sustainable pathway to enhance seed and protein yields while reducing reliance on synthetic mineral fertilizers in cool-climate regions.

## Figures and Tables

**Table 1:** Nodule number and nodule weight per plant, nitrogen stable isotope ratio, N content, and change in *δ*(^15^N) in different parts of soybean plants at flowering stage (R2).

Treatment	Nodule Number (Plant^−1^)	Nodule DW (g Plant^−1^)	Root		Shoot		Root to Shoot
			*δ*(^15^N), ‰	N, %	*δ*(^15^N), ‰	N, %	*Δ*(^15^N), ‰
M/Control			1.4 ± 0.7 ^a^	2.4 ± 0.3 ^a^	4.0 ± 0.8 ^b^	2.4 ± 0.4 ^ab^	−2.6
M + Fertilizer			1.3 ± 0.8 ^a^	2.2 ± 0.2 ^ab^	2.9 ± 0.6 ^ab^	2.3 ± 0.4 ^a^	−1.6
M + *AGF78*	23.1 ± 8.2 ^b^	0.10 ± 0.06 ^a^	1.7 ± 0.8 ^a^	2.4 ± 0.3 ^a^	2.8 ± 0.5 ^ab^	2.8 ± 0.3 ^ab^	−1.1
M + *SEMIA5079*	18.8 ± 7.1 ^ab^	0.09 ± 0.03 ^a^	1.3 ± 0.7 ^a^	2.1 ± 0.1 ^ab^	2.7 ± 0.6 ^a^	2.8 ± 0.2 ^ab^	−1.4
M + *RF10*	9.7 ± 9.3 ^ab^	0.06 ± 0.04 ^a^	2.3 ± 0.8 ^a^	1.6 ± 0.2 ^bc^	4.0 ± 0.5 ^b^	3.0 ± 0.3 ^b^	−1.7
L/Control			1.4 ± 0.8 ^a^	1.7 ± 0.2 ^bc^	3.0 ± 0.3 ^ab^	2.4 ± 0.2 ^ab^	−1.6
L + Fertilizer			1.8 ± 1.0 ^a^	1.2 ± 0.1 ^c^	2.9 ± 0.6 ^ab^	2.3 ± 0.2 ^ab^	−1.1
L + *AGF78*	13.0 ± 4.6 ^ab^	0.07 ± 0.03 ^a^	1.6 ± 0.9 ^a^	1.6 ± 0.3 ^bc^	2.7 ± 0.6 ^a^	2.9 ± 0.4 ^ab^	−1.1
L + *SEMIA5079*	11.2 ± 2.6 ^ab^	0.07 ± 0.03 ^a^	1.3 ± 0.6 ^a^	2.1 ± 0.2 ^ab^	2.5 ± 0.4 ^a^	2.8 ± 0.5 ^ab^	−1.2
L *+ RF10*	6.2 ± 3.7 ^a^	0.10 ± 0.05 ^a^	2.0 ± 0.6 ^a^	1.3 ± 0.4 ^c^	3.3 ± 0.6 ^ab^	2.5 ± 0.4 ^ab^	−1.3

Means followed by the same letters in the same column do not differ significantly from one another (one-way ANOVA, *p* ≤ 0.05). M refers to Merlin, L refers to Laulema, DW refers to dry weight.

**Table 2:** *δ*(^15^N) and N content and isotopic discrimination in different parts of soybean plants at full-maturity stage (R8).

Treatment	Root		Stem and Pod		Root−Stem/Pod	Seed		Root−Seed
	*δ*(^15^N), ‰	N, %	*δ*(^15^N), ‰	N, %	*Δ*(^15^N), ‰	*δ*(^15^N), ‰	N, %	*Δ*(^15^N), ‰
M/Control	2.2 ± 1.1 ^a^	0.6 ± 0.2 ^ab^	1.5 ± 0.4 ^ab^	0.5 ± 0.1 ^ab^	−0.7	2.4 ± 0.2 ^abe^	5.0 ± 0.4 ^a^	+0.2
M + Fertilizer	2.8 ± 0.6 ^ab^	0.7 ± 0.04 ^ab^	1.6 ± 0.4 ^ab^	0.5 ± 0.03 ^ab^	−1.2	2.1 ± 0.3 ^ab^	5.2 ±0.5 ^a^	−0.7
M + *AGF78*	3.5 ± 0.3 ^abc^	0.6 ± 0.1 ^ab^	0.8 ± 0.2 ^cd^	0.6 ± 0.04 ^ab^	−2.7	0.8 ± 0.4 ^cd^	6.6 ± 0.4 ^bc^	−2.7
M + *SEMIA5079*	3.5 ± 0.2 ^abc^	0.8 ± 0.1 ^ab^	0.0 ± 0.3 ^e^	0.6 ± 0.1 ^ab^	−3.5	0.7 ± 0.3 ^c^	6.7 ± 0.4 ^b^	−2.8
M + *RF10*	2.7 ± 0.2 ^ab^	0.7 ± 0.1 ^ab^	0.9 ± 0.4 ^acd^	0.6 ± 0.1 ^ab^	−1.8	0.8 ± 0.3 ^cd^	5.7 ± 0.3 ^acde^	−1.9
L/Control	2.0 ± 0.4 ^a^	0.5 ± 0.1 ^a^	1.2 ± 0.2 ^abc^	0.5 ± 0.04 ^a^	−0.8	2.5 ± 0.4 ^ae^	5.6 ± 0.3 ^ade^	+0.5
L + Fertilizer	2.6 ± 0.5 ^a^	0.7 ± 0.2 ^ab^	1.8 ± 0.3 ^b^	0.5 ± 0.03 ^ab^	−0.8	3.0 ± 0.2 ^e^	5.4 ± 0.4 ^ad^	+0.4
L + *AGF78*	4.1 ± 0.8 ^bc^	0.9 ± 0.03 ^bc^	0.6 ± 0.4 ^cde^	0.6 ± 0.1 ^b^	−3.5	1.1 ± 0.3 ^cdf^	6.3 ± 0.6 ^bcde^	−3.0
L + *SEMIA5079*	4.4 ± 0.9 ^c^	1.3 ± 0.4 ^c^	0.5 ± 0.3 ^de^	0.6 ± 0.1 ^ab^	−3.9	1.4 ± 0.2 ^df^	6.4 ± 0.4 ^bce^	−3.0
L *+ RF10*	3.0 ± 0.3 ^abc^	0.8 ± 0.1 ^bc^	0.8 ± 0.2 ^cde^	0.5 ± 0.1 ^ab^	−2.2	1.7 ± 0.1 ^bf^	5.9 ± 0.2 ^abcde^	−1.3

Means followed by the same letters in the same column do not differ significantly from one another (one-way ANOVA, *p* ≤ 0.05). M refers to Merlin, L refers to Laulema.

**Table 3:** Calculated nitrogen and carbon per hectare in different parts of soybean plants at flowering stage (R2).

Treatment	kg DW N ha^−1^		kg DW C ha^−1^	
	Root	Shoot	Root	Shoot
M/Control	19.7 ± 3.85 ^ab^	118 ± 16 ^a^	291 ± 33 ^a^	2211 ± 219 ^a^
M + Fertilizer	18.0 ± 3.46 ^abc^	125 ± 50 ^a^	299 ± 81 ^a^	2312 ± 797 ^a^
M + *AGF78*	23.8 ± 6.05 ^b^	198 ± 96 ^a^	384 ± 148 ^a^	2942 ± 1160 ^a^
M + *SEMIA5079*	23.1 ± 5.80 ^ab^	187 ± 65 ^a^	344 ± 62 ^a^	2837 ± 717 ^a^
M + *RF10*	17.3 ± 7.14 ^abc^	210 ± 84 ^a^	408 ± 117 ^a^	3013 ± 1187 ^a^
L/Control	15.0 ± 3.57 ^abc^	211 ± 70 ^a^	422 ± 126 ^a^	3180 ± 1302 ^a^
L + Fertilizer	7.21 ± 2.59 ^c^	145 ± 75 ^a^	260 ± 94 ^a^	3255 ± 1144 ^a^
L + *AGF78*	12.4 ± 4.00 ^ac^	154 ± 66 ^a^	307 ± 37 ^a^	2396 ± 1098 ^a^
L + *SEMIA5079*	15.3 ± 1.66 ^abc^	183 ± 67 ^a^	305 ± 56 ^a^	2817 ± 814 ^a^
L *+ RF10*	8.16 ± 0.81 ^c^	136 ± 29 ^a^	263 ± 58 ^a^	2452 ± 707 ^a^

Means followed by the same letters in the same column do not differ significantly from one another (one-way ANOVA, *p* ≤ 0.05). M refers to Merlin, L refers to Laulema, DW refers to dry weight.

**Table 4:** Calculated nitrogen and carbon per hectare in different parts of soybean plants at maturity stage (R8).

Treatment	kg DW N ha^−1^			kg DW C ha^−1^		
	Root	Stem and Pod	Seed	Root	Stem and Pod	Seed
M/Control	4.94 ± 1.88 ^a^	18.8 ± 4.70 ^a^	195.86 ± 46.98 ^a^	330 ± 65 ^ab^	1234 ± 864 ^b^	1944 ± 504 ^a^
M + Fertilizer	6.95 ± 1.46 ^a^	26.2 ± 5.80 ^a^	229.49 ± 59.90 ^a^	335 ± 54 ^ab^	2125 ± 401 ^ab^	2215 ± 734 ^a^
M + *AGF78*	6.03 ± 1.34 ^a^	35.8 ± 8.85 ^a^	486.64 ± 160.11 ^b^	375 ± 120 ^a^	2613 ± 655 ^ab^	3561 ± 1006 ^a^
M + *SEMIA5079*	6.10 ± 0.46 ^a^	28.0 ± 1.98 ^a^	374.76 ± 50.89 ^ab^	301 ± 25 ^ab^	2107 ± 385 ^ab^	2791 ± 388 ^a^
M + *RF10*	6.95 ± 1.22 ^a^	30.1 ± 5.94 ^a^	347.61 ± 72.06 ^ab^	372 ± 67 ^a^	2523 ± 329 ^ab^	3040 ± 745 ^a^
L/Control	4.47 ± 0.40 ^a^	33.3 ± 8.57 ^a^	339.60 ± 113.35 ^ab^	338 ± 53 ^ab^	3174 ± 724 ^a^	2909 ± 848 ^a^
L + Fertilizer	4.73 ± 0.93 ^a^	27.9 ± 4.82 ^a^	228.42 ± 62.20 ^a^	277 ± 45 ^ab^	2211 ± 294 ^ab^	2022 ± 438 ^a^
L + *AGF78*	6.14 ± 0.46 ^a^	35.8 ± 6.22 ^a^	326.88 ± 28.47 ^ab^	268 ± 22 ^ab^	2285 ± 454 ^ab^	2505 ± 406 ^a^
L + *SEMIA5079*	5.71 ± 4.67 ^a^	29.1 ± 9.53 ^a^	323.80 ± 107.36 ^ab^	170 ± 124 ^b^	2237 ± 984 ^ab^	2528 ± 863 ^a^
L *+ RF10*	4.95 ± 1.90 ^a^	30.4 ± 6.52 ^a^	286.68 ± 67.08 ^ab^	267 ± 79 ^ab^	2388 ± 502 ^ab^	2429 ± 575 ^a^

Means followed by the same letters in the same column do not differ significantly from one another (one-way ANOVA, *p* ≤ 0.05). M refers to Merlin, L refers to Laulema, DW refers to dry weight.

**Table 5:** Carbon stable isotope ratio, carbon content and C/N ratio in roots and shoots at flowering stage (R2).

Treatment		Root			Shoot	
	*δ*(^13^C), ‰	C, %	C/N	*δ*(^13^C), ‰	C, %	C/N
M/Control	–27.0 ± 0.3 ^a^	35.7 ± 1.2 ^bc^	15.0 ± 2.5 ^b^	–27.0 ± 0.4 ^ab^	41.9 ± 1.1 ^a^	18.8 ± 1.2 ^bc^
M + Fertilizer	–27.0 ± 0. 5 ^a^	36.0 ± 1.6 ^abc^	16.5 ± 1.6 ^ab^	–26.8 ± 0.2 ^ab^	42.5 ± 1.1 ^a^	17.4 ± 1.4 ^abc^
M + *AGF78*	–26.8 ± 0.4 ^a^	36.6 ± 2.9 ^abc^	15.7 ± 2.3 ^ab^	–27.0 ± 0.5 ^ab^	42.7 ± 0.7 ^a^	15.6 ± 1.7 ^abc^
M + *SEMIA5079*	–26.8 ± 0.3 ^a^	32.1 ± 2.2 ^b^	15.1 ± 1.7 ^ab^	–26.9 ± 0.3 ^ab^	42.4 ± 1.1 ^a^	15.4 ± 1.3 ^ab^
M + *RF10*	–26.7 ± 0.2 ^a^	39.6 ± 1.7 ^ac^	24.7 ± 4.4 ^abc^	–27.1 ± 0.2 ^a^	44.0 ± 2.7 ^a^	14.5 ± 0.6 ^a^
L/Control	–26.8 ± 0.7 ^a^	41.2 ± 2.3 ^ac^	23.8 ± 4.2 ^abcd^	–25.9 ± 0.2 ^c^	42.8 ± 0.4 ^a^	17.9 ± 1.7 ^abc^
L + Fertilizer	–26.6 ± 0.2 ^a^	41.2 ± 0.3 ^a^	36.2 ± 3. 7 ^d^	–26.4 ± 0.5 ^abc^	42.8 ± 0.6 ^a^	19.3 ± 0.9 ^c^
L + *AGF78*	–26.7 ± 0.4 ^a^	40.6 ± 0.8 ^ac^	25.9 ± 5.9 ^acd^	–26.2 ± 0.3 ^bc^	43.3 ± 0.7 ^a^	15.3 ± 1.9 ^ab^
L + *SEMIA5079*	–26.3 ± 0.2 ^a^	40.4 ± 3.2 ^ac^	19.8 ± 2.7 ^ab^	–26.3 ± 0.4 ^abc^	43.0 ± 0.3 ^a^	15.9 ± 2.4 ^abc^
L *+ RF10*	–26.4 ± 0.4 ^a^	41.1 ± 1.4 ^a^	32.5 ± 8.2 ^cd^	–26.3 ± 0.5 ^abc^	42.9 ± 0.4 ^a^	15.2 ± 1.3 ^abc^

Means followed by the same letters in the same column do not differ significantly from one another (one-way ANOVA, *p* ≤ 0.05). M refers to Merlin, L refers to Laulema.

**Table 6:** Carbon stable isotope ratio, carbon content and C/N ratio in roots, stems and pods and seeds at maturity (R8).

Treatment		Root		Stem and Pod				Seed	
	*δ*(^13^C), ‰	C, %	C/N	*δ*(^13^C), ‰	C, %	C/N	*δ*(^13^C), ‰	C, %	C/N
M/Control	–26.2 ± 0.4 ^ac^	39.4 ± 1.8 ^bcd^	70.8 ± 14.2 ^bc^	–26.6 ± 0.2 ^ab^	41.9 ± 3.3 ^a^	88.2 ± 4.5 ^bc^	–26.8 ± 0.2 ^abcd^	49.4 ± 1.4 ^a^	9.2 ± 0.2 ^abc^
M + Fertilizer	–25.9 ± 0.3 ^abcd^	35.3 ± 1.0 ^ab^	48.6 ± 2.9 ^abc^	–26.6 ± 0.3 ^ab^	40.0 ± 0.5 ^a^	81.6 ± 3.7 ^abc^	–26.8 ± 0.2 ^abcd^	49.5 ± 1.3 ^a^	9.6 ± 1.1 ^a^
M + *AGF78*	–26.2 ± 0.3 ^abc^	36.7 ± 1.0 ^abc^	61.1 ± 6.8 ^abc^	–26.4 ± 0.5 ^ac^	40.3 ± 0.3 ^a^	73.3 ± 4.9 ^abd^	–27.7 ± 0. 5 ^a^	48.8 ± 0.5 ^a^	7.4 ± 0.5 ^b^
M + *SEMIA5079*	–26.3 ± 0.2 ^c^	36.7 ± 2.1 ^abc^	49.5 ± 4.8 ^abc^	–26.6 ± 0.4 ^ab^	42.4 ± 3.5 ^a^	69.4 ± 8.1 ^ad^	–27.4 ± 0.3 ^abc^	49.8 ± 1.8 ^a^	7.5 ± 0.4 ^b^
M + *RF10*	–26.1 ± 0.4 ^abc^	35.0 ± 2.7 ^a^	54.8 ± 12.6 ^abc^	–27.2 ± 0.3 ^b^	41.5 ± 2.1 ^a^	84.6 ± 6.6 ^abc^	–27.7 ± 0.3 ^ac^	49.6 ± 3.3 ^a^	8.7 ± 0.7 ^abc^
L/Control	–25.0 ± 0.2 ^e^	40.6 ± 0.7 ^cd^	75.7 ± 9.6 ^b^	–25.9 ± 0.2 ^acd^	43.9 ± 2.9 ^a^	95.9 ± 3.8 ^c^	–26.4 ± 0.3 ^bd^	48.4 ± 0.8 ^a^	8.7 ± 0.6 ^abc^
L + Fertilizer	–25.5 ± 0.3 ^abde^	39.3 ± 2.7 ^abcd^	61.4 ± 21.1 ^abc^	–26.3 ± 0.2 ^ac^	40.0 ± 0.4 ^a^	79.9 ± 4.9 ^ab^	–26.6 ± 0.4 ^bcd^	48.1 ± 1.9 ^a^	8.9 ± 0.5 ^ac^
L + *AGF78*	–25.3 ± 0.3 ^de^	40.1 ± 0.8 ^cd^	43.7 ± 1.2 ^ac^	–25.7 ± 0.2 ^cd^	40.3 ± 1.0 ^a^	60.1 ± 9.3 ^d^	–25.8 ± 0.5 ^d^	48.0 ± 0.9 ^a^	7.6 ± 0.6 ^bc^
L + *SEMIA5079*	–25.4 ± 0.4 ^bde^	38.6 ± 2.6 ^abcd^	32.4 ± 13.2 ^a^	–25.5 ± 0.3 ^d^	40.6 ± 1.0 ^a^	66.3 ± 8.8 ^ad^	–26.7 ± 0.9 ^abcd^	49.7 ± 1.5 ^a^	7.8 ± 0.5 ^bc^
L *+ RF10*	–25.6 ± 0.3 ^abcde^	41.5 ± 1.3 ^d^	58.0 ± 11.6 ^abc^	–25.9 ± 0.5 ^acd^	41.3 ± 1.2 ^a^	74.2 ± 10.4 ^abd^	–26.3 ± 0.5 ^bd^	49.9 ± 1.1 ^a^	8.5 ± 0.4 ^abc^

Means followed by the same letters in the same column do not differ significantly from one another (one-way ANOVA, *p* ≤ 0.05). M refers to Merlin, L refers to Laulema.

**Table 7:** Agronomical parameters in inoculated and non-inoculated soybeans.

Treatment	Seed Yield, g m^−2^	1000-Seed Weight, g	Seed Protein, %
M/Control	202 ± 75 ^abc^	161 ± 16.8 ^a^	31.1 ± 2.75 ^ad^
M + Fertilizer	234 ± 66 ^abc^	158 ± 7.5 ^a^	30.5 ± 1.29 ^a^
M + *AGF78*	294 ± 55 ^ab^	176 ± 7.1 ^ab^	36.8 ± 0.29 ^b^
M + *SEMIA5079*	315 ± 74 ^b^	179 ± 12.0 ^ab^	36.9 ± 0.45 ^b^
M + *RF10*	261 ± 93 ^abc^	174 ± 12.0 ^ab^	33.8 ± 1.56 ^cd^
L/Control	153 ± 28 ^c^	194 ± 2.0 ^bc^	32.9 ± 0.42 ^acd^
L + Fertilizer	136 ± 30 ^c^	191 ± 5.6 ^bc^	32.6 ± 0.44 ^acd^
L + *AGF78*	178 ± 42 ^ac^	209 ± 3.2 ^c^	37.4 ± 0.20 ^b^
L + *SEMIA5079*	181 ± 30 ^abc^	206 ± 5.9 ^c^	36.7 ± 0.84 ^b^
L *+ RF10*	136 ± 20 ^c^	195 ± 4.1 ^bc^	34.9 ± 0.82 ^bc^

Means followed by the same letters in the same column do not differ significantly from one another (one-way ANOVA, *p* ≤ 0.05). M refers to Merlin, L refers to Laulema.

**Table 8:** Experimental design for inoculation with commercially available *B. japonicum* bacterial strains along with control and fertilization treatments.

Factor 1—Soybean Variety (Merlin—M or Laulema—L)			
Factor 2—Amendment			
No.	Amendment	Manufacturer	Abbreviation
1	Control (neither fertilization, nor inoculation)	No inoculation, no fertilizers	Control
2	Organic cattle manure	Granulated cattle manure fertilizer (N—3%, *δ*(^15^N)—6.5‰, P_2_O_5_—1.5%, K_2_O—7%, organic matter ~72%, pH 9), Agrolinija	Fertilizer
3	*B. japonicum* strain AGF_78	*Bactolive* RHIZO-MIC UG, St. Johann, Germany	AGF_78
4	*B. japonicum strain* SEMIA 5079	Basf HiStick HiStick^®^ Soy (BASF, Ludwigshafen, Germany)	SEMIA_5079
5	*B. japonicum* strain RF10	RhizoFix RF-10 Feldsaaten Freudenberger GmbH & Co. KGcontaining, Krefeld, Germany	RF_10
10 treatments as the combination of factors: (1) M/Control; (2) M + Fertilizer; (3) M + AGF78; (4) M + SEMIA5079; (5) M + RF10; (6) L/Control; (7) L + Fertilizer; (8) L + AGF78; (9) L + SEMIA5079; (10) L + RF10			

## Data Availability

The original contributions presented in this study are included in the article. Further inquiries can be directed to the corresponding author.
